# Reflexivity gradient—Consciousness knowing itself

**DOI:** 10.3389/fpsyg.2024.1450553

**Published:** 2024-08-23

**Authors:** Zoran Josipovic

**Affiliations:** ^1^Department of Psychology, New York University, New York, NY, United States; ^2^Nonduality Institute, Woodstock, NY, United States

**Keywords:** reflexivity gradient, nondual reflexivity, awareness of awareness, consciousness itself, nondual awareness

## Abstract

Some consider phenomenal consciousness to be the great achievement of the evolution of life on earth, but the real achievement is much more than mere phenomenality. The real achievement is that consciousness has woken up within us and has recognized itself, that within us humans, consciousness knows that it is conscious. This short review explores the reflexivity of consciousness from the perspective of consciousness itself—a non-conceptual nondual awareness, whose main property is its non-representational reflexivity. In light of this nondual reflexivity, different types of reflexivity proposed by current theories can be seen as a gradation of relational or transitive distances between consciousness as the knower and consciousness as the known, from fully representational and dual, through various forms of qualified monism, to fully non-representational and nondual.

## Introduction

Much of the current research on consciousness could be summed up by a well-known metaphor: standing outdoors on a sunny day while facing away from the sun, one may see various objects and events in the environment as illuminated by the light reflected off them and mistakenly conclude that they themselves are the original source of that light. Similarly, insisting on explaining consciousness as something other than itself, as a phenomenal content, a cognitive or affective function, a state of arousal, or a conceptual structure, we fail to see consciousness itself. Admittedly, these aspects are parts of conscious experiencing, and a great deal of progress has been made in recent years in understanding them (Koch et al., 2016; Michel et al., 2019; Lepauvre and Melloni, 2021). However, consciousness itself or consciousness as such—a foundational awareness that is distinct from contents, functions, and states—is still insufficiently researched.

Attempts to include it within contemporary discourse on consciousness are slowly gaining traction in neuroscience and the philosophy of mind (Josipovic, 2014, 2019, 2021; Ricard and Singer, 2017; Dunne et al., 2019; Metzinger, 2020, 2024) but are often plagued by misunderstandings of what this conscious is. In this research, as I have done over a number of years (Josipovic, 2014, 2016, 2019, 2021; Josipovic and Miskovic, 2020), I have presented the perspective that consciousness itself is a type of awareness whose main property is its inherent, non-representational reflexivity—it knows that it is aware without needing mediation by mental representations.

Consciousness itself does not rely on mental representations to know either itself or what is present to it; thus, it is a different way of knowing from the usual conceptual mind that is based on mental representations. This awareness is nondual, both within itself and between itself and phenomena. It is nondual within itself because it knows itself without taking itself as an object of this knowing, and it is nondual between itself and phenomena because it knows phenomena without taking itself as a separate conceptually reified subject and phenomena as its objects. This awareness does not fragment experiences into reified dualities of subject vs. object, self vs. other, us vs. them, good vs. bad, and similar. Hence, it has been termed nondual awareness (Williams, 2000; Higgins, 2013; Josipovic, 2014, 2021; Laish, 2015; MacKenzie, 2015).

Although a singular presence, when nondual awareness is explicit, its inherent properties become self-evident, irrespective of whether they are subsequently conceptualized or not. These aspects have been discussed in other studies (Rabjam, 2007; Josipovic, 2019, 2021; Fasching, 2021) and are only listed here in four groups: (1) Being, Presence, Emptiness, and Spaciousness; (2) Cognitive Luminosity, and Reflexivity; (3) Bliss, Ecstasy, Universal Love, and Compassion; and (4) Singularity, Unity, and Self.

Since nondual awareness is singular and uncompounded, its dimensions are not separate elements from which awareness is assembled or from whose relationships or interactions it emerges.

When present explicitly in an experience, nondual awareness appears as distinct from any phenomenal content that is co-present with it, from the functions that create content, and from global states, as well as from unconscious substrate that structures ordinary concept-based experiencing. Nondual awareness appears as that which is, as has been, conscious or aware in any experience; in other words, it appears as consciousness itself or consciousness as such.

However, ordinarily, although present, this awareness is only implicit in an experience but can become explicit under special circumstances or due to certain contemplative and other practices. Therefore, for any experience, there is a gradient of how implicit or explicit nondual awareness is in that experience, and that gradient is orthogonal to the local content and global state (for a detailed discussion see Josipovic, 2021).

When nondual awareness is fully explicit during wakefulness, it is experienced as simultaneously transcendent and immanent in conscious states and contents. It is transcendent, as the silent aware space that pervades and encompasses the entire conscious experience, one's entire perceptual bubble, and it is immanent, as that out of which everything is made, the way water in a glass is both the medium in which ice cubes float and the substance out of which they are made (Josipovic, 2016, 2021).

As stated above, the main property of consciousness itself or nondual awareness is its inherent, non-representational reflexivity—it knows that it is aware without needing mediation by mental representations and without taking itself as an intentional object; hence, it is non-relational. This type of reflexivity can be termed as nondual reflexivity. It is unique to consciousness itself and is that which makes consciousness itself what it is. The implicit–explicit gradient of nondual awareness can be understood as the gradient of how evident nondual awareness is to itself, or in other words, as the gradient of its reflexivity (Josipovic, 2021).

I have discussed in detail the neural correlates of nondual awareness and non-representational nondual reflexivity previously (Josipovic, 2019, 2021). Presently, I will only briefly summarize them in order to further clarify the present discussion.

Although isomorphism between phenomenal and neural levels should not be presumed, neither should it be rejected *a priori*. Since nondual awareness is phenomenally and functionally distinct from attention, monitoring, working memory, evaluation, and decision, i.e., from processes that contribute to constructing perceptual, affective, and cognitive contents and from those that determine global states, its neural correlate could likewise be distinct—a dedicated network with its characteristic dynamics. A neural correlate of nondual awareness needs to be able to function with both low and high levels of arousal and amounts of content and serve as the integrative conscious space within which both intrinsic and extrinsic contents can co-occur.

I have previously proposed that the central precuneus network with a self-sustaining oscillatory resonant dynamic regime is the most likely neural correlate of nondual awareness (Josipovic, 2014, 2019, 2021). This functional network links the central precuneus with the dorsolateral prefrontal cortex, the dorsal anterior cingulate cortex, the dorsomedial prefrontal cortex, and the inferior parietal lobule (Cavanna and Trimble, 2006; Margulies et al., 2009; Cunningham et al., 2017; Buckner and DiNicola, 2019). The central precuneus is unique among different subdivisions of the precuneus as it can functionally connect with both the intrinsic (default mode network) and the extrinsic (dorsal attention network and executive control network) systems (Li et al., 2019). This finding corresponds to a major function of nondual awareness in increasing the integration of intrinsic self-related and extrinsic environment-related aspects of experience (Josipovic et al., 2012; Josipovic, 2014). This role functionally differentiates the neural correlate of nondual awareness from the neural correlate of monitoring, which is associated with networks for salience detection and involuntary attention, whose effect is to induce switching between the intrinsic and extrinsic systems in the brain and increase their functional segregation (Josipovic, 2010, 2013, 2014, 2019). Like other cortical networks, the central precuneus network is reciprocally connected to the subcortical nuclei of the reticular activating system that supply arousal and to the thalamic nuclei that enable its cortical organization (Tomasi and Volkow, 2011). However, these sub-cortical areas, although necessary, are not sufficient by themselves for nondual awareness.

When nondual awareness is explicit during normal wakefulness and its inherent reflexivity is vividly present, the pre-frontal nodes of its network, the dorsolateral prefrontal cortex in particular, function to add the necessary amplitude and persistence to the network-wide resonance and coherence (Helfrich and Knight, 2016; Schmidt et al., 2018). On the other hand, it is possible, especially at times of minimized phenomenal content, that an increased level of functional integration or recursive feedback in the posterior nodes of the central precuneus network is alone sufficient to establish sustained oscillatory resonance, in which neurons inform each other about their excitation levels, or in other words, their information processing capacity, without processing any other content, which is experienced as inherent, non-representational nondual reflexivity. Furthermore, a neural network informing itself about its capacity to process information can be instituted in a relatively simple electronic circuit, without any sign of awareness or consciousness. Hence, the biological constraints on a system's capacity for consciousness apply here and, even more importantly, for nondual awareness that requires a human-level brain (Josipovic, 2021).

In light of nondual reflexivity, different types of reflexivity proposed by current theories can be seen as a gradation of relational or transitive distances between consciousness as the knower and consciousness as the known, ranging from fully representational and dual, through various forms of qualified monism, to fully non-representational and nondual. The types of reflexivity are shown in Table 1.

**Table 1:** Reflexivity gradient—consciousness knowing itself.

**Implicit**	**Transitional**	**Explicit**
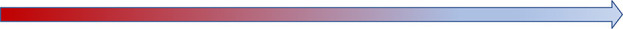		
**Dual**	**Qualified monist**	**Nondual**
Representational	Representational or non-representational	Non-representational
Conceptual	Conceptual or non-conceptual	Non-conceptual
Relational transitive	Relational or non-relational	Non-relational

Reflexivity gradient with three zones indicating transitive or relational distance: dual, qualified monist, and nondual, corresponding to the three zones of the implicit-explicit gradient of nondual awareness: implicit, transitional, and explicit. Dualistic reflexivity is representational, conceptual, and transitive, with the different-order knower and known. Qualified monist reflexivity can be either representational or non-representational, conceptual or non-conceptual, and relational or non-relational, with the same-order knower and known. Nondual reflexivity is non-representational, non-conceptual, and non-relational, without the knower–known structure.

## Reflexivity theories

Theories of reflexivity have been previously grouped broadly into two types, based largely on how they view the nature and the role of representation in consciousness (Siewert, 2022). The first type can be termed the mental representational or cognitive-analytic type, and it holds that, for an experience or a mental state to be conscious, it has to be represented by another state that is different from it (Rosenthal, 2004, 2012; Gennaro, 2013). This idea is known as the transitivity principle (ibid.) and indicates a relationship between two kinds of mental representations, those representing the state itself, that is, what we are conscious of, and those re-representing them, that is, how we are conscious of it. In cases of reflexivity, this transitivity principle necessitates a third-order representation, re-representing the second one (Rosenthal, 2012).

The other type of reflexivity theories are the phenomenological theories, which can be intentional representational, or non-intentional (Zahavi, 2005; Montague, 2016; Gallagher, 2022; Strawson, 2022). Here, representation is understood as phenomenal intention of conscious states, their about-ness. Reflexivity is seen as a more immediate self-knowing that accompanies most, if not all, conscious states and is pre-reflectively “given” with experience, not requiring reflection or mental re-representation; hence, it is not explicitly transitive. For most phenomenologists, consciousness knowing itself is pre-reflective self-knowing, which is understood as not explicitly conscious but as nevertheless present and enabling all conscious experiences.

From the viewpoint of nondual awareness, these two types of theories can be seen as reflecting the two seemingly contradictory aspects of nondual awareness: its transcendence and its immanence. In their claim that representations that give rise to conscious knowing are of a different order than those that represent what we are conscious of, representational theories reflect the transcendent aspect of nondual awareness, the fact that consciousness itself is distinct from all other aspects of experience, the way space is different from everything in it. In their claim that reflexive knowing is intrinsic to experience, phenomenological theories reflect the immanent aspect of consciousness itself, where nondual awareness appears as the substance out of which everything in experience is made, the way water is the substance out of which ice cubes that float in it are made.

Representational and phenomenological theories also differ in terms of the epistemic distance they propose between the knower and the known. Representational theories are more indirect in that re-representations needed for conscious knowing, in general, and especially for reflexivity, in particular, are of an entirely different order from those needed to represent that which is known. Conscious knowing is seen as a relational property conferred onto the known by these higher-order representations (Lycan, 2023). Phenomenological theories, on the other hand, are more direct. They reject the idea that the states that one is conscious of are objects of consciousness. Likewise, they do not agree that, in reflexivity, consciousness is conscious of itself as its object (Zahavi, 2005, 2018). In other words, they do not accept the epistemic dualism of the subject as the knower and the object as the known. Instead, they propose that what makes an experience, in general, conscious is intrinsic to that conscious experience and that reflexivity is an inherent and even defining property of consciousness itself, requiring neither a higher-order nor same-order representation (Gallagher and Zahavi, 2023).

## Dual representational

### Strong transparency

Reductive representationalism sees consciousness as entirely reducible to mental representations (Lycan, 2023). When such representations related to the subject and object are strongly reified, phenomenal consciousness can appear to be just an illusion and consciousness knowing itself, an impossibility. The idea that the mind cannot know itself is an old one, appearing first in the Brihadaranyaka Upanishad (Radhakrishnan, 1994) and later in various Buddhist sutras (Luk, 2001). Briefly, it could be said that it applies only to the impossibility of knowing consciousness itself via dualistic conceptual thinking (Sansk. vijnana) but does not hold true for knowing more directly via intuitive awareness (Sansk. prajnana), or in other words, via intrinsic reflexivity of consciousness itself.

In Western philosophy, ideas about the impossibility of the mind knowing itself have been expressed most clearly by Hume (1978) and more recently by Tye (2014) and Dennett (1987), and in the context of cognitive neuroscience, the ideas have been expressed by Frankish (2016). The more recent views are sometimes referred to as strong transparency arguments or as reductive representationalism (Dretske, 1995; Tye, 2014).

According to these, any attempt to introspect consciousness finds only properties of objects in external or internal environments, but not the actual phenomenal qualities of experience, nor the consciousness itself, since on this account, representational processes such as spatial perspective, body-ownership, and agency, that are involved in the minimal or core self and confer these properties to subjective phenomenality, are unconscious and not available to introspection. In the well-known metaphor, they are like a highly transparent windowpane that one looks through, but which one does not itself see (Metzinger, 2010).

The strong transparency thesis has been argued against extensively by many (Zahavi, 2005; Montague, 2016; Chalmers, 2020), so I will not explore those arguments in the present discourse. I will only make a couple of points from the perspective of nondual awareness, and contemplative practice more generally. These offer two different ways to notice the usually unconscious processes involved in constructing minimal self experience or even the homeostatic proto-self identity, in addition to relatively common ‘seeing-through' and de-constructing of various extended and narrative self-models. One is through developing ability to sustain focused attention for prolonged periods of time, resulting in various absorption states where, for however brief periods, there is cessation of these minimal self processes, followed by their reappearance once one emerges from the absorption state (Josipovic and Miskovic, 2020; Metzinger, 2024). Alternatively, once nondual awareness is discovered and stabilized, one can, at times, become aware of such processes because this awareness is, phenomenally, the most subtle aspect of conscious experience. These and other processes involved in constructing the self and environment then appear to it as contents in its epistemic space (Josipovic, 2021).

The claimed inability of introspection to find anything other than external phenomenal contents under ordinary dualistic cognition (Tye, 2014; Montague, 2016) is, in part, due to attention being habitually oriented toward finding and attending to an object. In other words, the abovementioned claim is due to the inability to sufficiently turn the attention around to attend to awareness itself (Josipovic and Miskovic, 2020). This “turning around” is not some act of permanent contortion but is meant to instigate a collapse of the dualistic attending and monitoring into just being aware and, in doing so, reveal nondual awareness—consciousness itself— as already present in one's experience.

## Dual representational

### Higher order

Unlike reductive representation theories, the less reductive or even non-reductive representational theories allow for the possibility of consciousness knowing itself (Rosenthal, 2004; Carruthers and Gennaro, 2023). In terms of transitivity distance, these theories are, at least in their main forms, also strongly dualistic.

According to higher-order theories, conscious experiencing is possible because the first-order representations of some contents or states, which are themselves unconscious, are re-represented by certain higher-order representations that are different from them (Rosenthal, 2004, 2012). In other words, the first-order representations are the objects to which higher-order representations are directed. Higher-order representations are generally understood as enabling access consciousness or as being equivalent to it (Block, 2007) or as being a function of monitoring (Brown et al., 2019; Lycan, 2023). The phenomenal properties of experience are believed to be the semantic properties of these higher-order representations (Siewert, 2022). The most recent version of a higher-order theory, Brown's higher-order representation of representation theory as applied to emotions by LeDoux (Brown et al., 2019; LeDoux, 2024), points to a hierarchy of higher-order representations, which results in a gradation of conscious experience from pre-conscious to fully conscious, i.e., from anoetic to noetic and autonoetic.

Higher-order representations responsible for the conscious state or inner awareness are themselves unconscious, non-inferential, and not available for direct introspection (Rosenthal, 2004, 2012). However, when reflecting on one's experience, such as during confidence judgments (Webb et al., 2023), inferential decisions (Fleming, 2020), or conceptual introspection (Carruthers and Gennaro, 2023), they or their re-representations become fully conscious metacognition. Reflexivity, and especially being aware that one is aware, is then due to a third-order re-representation of those higher-order representations. According to this view, it occurs only in conscious introspection that requires focusing attention on some conscious states (Rosenthal, 2012). From the perspective of nondual awareness, the necessity of a third-order re-representation for awareness of awareness seems like an obvious indication that such conceptual processes cannot be the mechanism of inherent reflexivity of consciousness itself. Since nondual reflexivity is, so to speak, immediate, as an inherent property of awareness, and is non-conceptual and non-transitive, phenomenally, it is very different from attending as a subject to awareness as an object of one's conceptual introspecting (Josipovic, 2019, 2021).

Some higher-order theorists have proposed that, in a transition to conscious metacognitive states, higher-order representations themselves shift from being unconscious to being conscious (Gennaro, 2013). This shift has raised questions of how conscious experience can come from two equally unconscious representations; how an infinite regression of re-representations can be avoided; and what causes higher-order representations to shift from being unconscious to being conscious (Zahavi, 2005; Kriegel, 2009; Montague, 2016).

Different variants of higher-order theories could be seen, in addition to their main differences as also differing in terms of transitivity distance between their higher-order representations and that which they represent. For example, higher-order perception theories for which higher-order representations are perception-like outputs of internal monitoring could be considered less dualistic than the assertoric meta-thoughts of a higher-order thought theory (Carruthers and Gennaro, 2023). Similarly, a higher-order global state theory for which a higher-order representation is a global self-world representational state, which encompasses first-order representations, could be seen as arguably less dualistic (Van Gulick, 2004). The wide intrinsicality view (Gennaro, 2013; Cole, 2014) in which a higher-order representation is intrinsic to the first-order state it represents is even less dualistic and, in the intrinsicality claim, it begins to resemble those qualified monist theories of reflexivity that reject the higher-order premise altogether.

## Qualified monist

### Self-representation

Discomfort with the dualistic transitive distance between higher-order representations and what they re-represent can be seen as motivating the same-order representational theories that are both representational and relational but claim that consciousness knowing itself is a special kind of relationship. Hence, they could be termed as qualified monist theories (Kriegel, 2009; Montague, 2016; Strawson, 2022).

Self-representation theory (Kriegel, 2009) claims that a mental state that is conscious represents itself but is one with that self-representation. Conscious experience is then seen as an integration of representations for content properties—what is being conscious, with representations for subjective character—representing-as-occurring-now-in-me (Kriegel, 2024). Self-representation responsible for the reflexive property of experience, also known as inner awareness, is not a type of thought or a type of perception resulting from monitoring, as higher-order theories claim, but a unique kind of representation that is more intimate. In other words, self-representation responsible for the reflexive property of experience is less dualistic and yet still relational as consciousness takes itself as its object. Furthermore, on this view, it is only in virtue of such self-representations that a state is phenomenally conscious (Kriegel, 2024).

Same-order approaches have been criticized on similar grounds as the higher-order ones, that they still contain the problem of how to make the two representations, for content properties and for subjective character, one unified experience (Zahavi, 2018). In addition, intentional representations are seen as not being able to reference themselves reflexively since the direction of intentionality of consciousness, according to phenomenological orthodoxy, is always away from itself and toward something other than itself (Peters, 2013).

## Qualified monist

### Objectivist

Reflexivity theories in this group are largely phenomenological and perceive reflexivity as representational and relational; unlike dualist or qualified dualist, they think of reflexive act as being “implicit” in, or given with, any conscious experience (Montague, 2016; Strawson, 2022). Theories in this group hold that reflexivity does not require consciousness to consider itself a separate object of introspective reflection in order to know itself. This further step toward decreasing the distance between consciousness as the knower and consciousness as the known could be seen as a jump to a different level of cognitive intimacy from the previous ones, as here, the two, though still different, are given together within one experience, as a symbiosis of sorts. Hence, these theories can be thought of as different versions of qualified monism.

According to an early version of this view attributed to Brentano (Gallagher, 2022) and other similar same-order representation views, any experience is constituted by two simultaneous components: awareness of its content, whether perceptual, affective, or cognitive; and an awareness that perceiving, feeling, etc., is occurring, or in other words, a reflexive awareness that one is having a particular conscious experience. These two make one mental state and one unified experience. In terms of intentionality, this implies that, within a single experience, intention is divided into two co-occurring and related intentions, one oriented toward the content and the other directed reflexively toward itself. These two intentional targets have been considered the primary and the secondary objects of consciousness, giving these views their characteristic objectivist orientation (Gallagher and Zahavi, 2023). With respect to reflexivity, they also indicate a relational gap between awareness as the knower and awareness as the known.

By defining representation very broadly as intentionality or about-ness with respect to anything that is experienced and side-stepping the older argument over whether intentionality is noetic or noematic, contemporary interpretations of the above view of reflexivity (Montague, 2016) claim that all phenomenal contents are representational, in the sense that, with any conscious experience, there is something that is being experienced. In the same spirit, reflexivity is also seen as representational and relational since, minimally, it is about being aware that one is aware (Montague, 2017). This view then allows for the redefining of the relational gap between awareness as representation and awareness as the one that is being represented. This is something that has posed a problem for early phenomenologists such as Brentano and Husserl (Zahavi, 2005). The claim is then that reflexivity, which is given with any experience, does not contain a subject–object gap between the knower and the known. Nevertheless, by insisting on the relational nature of reflexivity, they could not come any closer to an explanation of it than to restate a view held by the self-representation theory, which attributes this absence of gap to awareness' relation to itself being somehow special due to the uniqueness of consciousness (Montague, 2017).

From the viewpoint of nondual awareness or consciousness as such, these observations can be regarded as accurate intuitions arising from awareness itself but which are then being distorted by unconscious conceptual reifications and relational concepts. In other words, at a representational level, being conscious that we are consciously experiencing is a derivative of the inherent non-representational reflexivity of consciousness itself. As a result, these theories fall short in explanatory power as they do not yet recognize non-representational nondual awareness as foundational consciousness or consciousness itself for which reflexivity is not a relation but its intrinsic property.

Without discovering nondual awareness and its non-conceptual and non-representational mode of knowing, it will remain difficult for an objectivist approach to understand how one can know the nature of reflexivity since a more direct reflexivity cannot be conceptually introspected as a separate state or object (Montague, 2016; Josipovic, 2019). Similarly, without stabilizing nondual awareness in an ordinary waking experience, it can be difficult to see how it is possible to experience the properties of awareness itself, including nondual reflexivity, as distinct from the phenomenal properties of perceptual and other contents. Since nondual awareness can, in principle, pervade and encompass all other aspects of experience, including conceptual processes, ordinary introspection can occur within the epistemic space of nondual awareness as just another type of a cognitive event.

## Qualified monist

### Subjectivist

Reflexivity theories in this group see reflexivity as non-relational and non-intentional, and instead, as a property of conscious subject or some minimal phenomenal self that is present in any experience (Zahavi, 2005, 2018, 2024; Gallagher, 2022; Marchetti, 2024). They argue that any intentional stance toward consciousness is necessarily objectifying and that one is already pre-reflectively self-aware without having to become one's intentional object (Zahavi, 2005; Frank, 2007). Furthermore, any objectifying intentional conscious state is claimed to have an underlying pre-reflective, non-relational reflexivity that makes it possible. It can then be said that these views are basically monist as they view all experience as subjective experience.

Unlike noematic intentionality, reflexivity in these more recent subjectivist theories is non-perspectival and does not require an observational distance and perspective from which a subject is witnessing experience and awareness (Zahavi, 2005). Instead of intentionality, they propose that self-awareness implies identity and that consciousness is intrinsically self-aware.

A question has been raised that, if self-awareness is non-representational, how is it then instantiated (Montague, 2016)? One view sees intrinsic reflexivity not only as non-intentional and non-relational but also, somewhat contradictorily, as dependent on mental representations, for example, as a schema of a system's capacity to represent (Peters, 2013). Another view sees self-awareness as having a unique temporal structure, one that is distinct from that of intentional consciousness and especially from that of fully conceptual reflective introspection. Husserl (1913) termed this structure as an impression-retention-protention structure, indicating something akin to an experience enabled by short-term memory only, which can track, retain, and make predictions or expectations over short time scales (Zahavi, 2003). Pre-reflective self-awareness is, on this view, unified with whatever phenomena appear with it in an experience.

As previously mentioned, these theories correctly intuit the immanence of consciousness itself in and as experience. However, they do not see its transcendence and, hence, do not understand its space as the unchanging ground of being. This problem is in part due to the implicit serial view of experiencing. Temporal views always involve some, however subtle, subject–object dualities of the attender and the attended, and the observer and the observed, where the observed stream of consciousness unfolds as a series of successive events. Reflexivity, even if only viewed as pre-reflective self-awareness, is seen as a temporal process that unfolds over a time span, however short in duration. In contrast, consciousness itself or nondual awareness is atemporally present, but this should not be understood from a temporal perspective as that would lead to the impossibility of instantaneousness. Rather, the correct perspective here is spatial, as nondual awareness is present to itself all at once, the way space is phenomenally an all-encompassing steady background within which things and events occur (Blackstone, 2007; Josipovic, 2019, 2021). Therefore, in respect to phenomena, it does not have a separate attender who, for example, attends to a melody. Rather, a melody simply occurs within its space. In addition, since nondual awareness knows by merely mirroring, there is no transitive distance between this awareness and the phenomena that appear to it, akin to the way images that are reflected in a mirror are different from the mirror but are not separate from it (Josipovic, 2021). Furthermore, the impression-retention-protention structure indicates a certain degree of mental representations, which are not intrinsic to consciousness itself but can co-occur within it as structures and events within its intrinsically empty epistemic space.

## Nondual reflexivity

Nondual reflexivity is the inherent property of consciousness itself and entirely non-representational and non-intentional, or in other words, nondual. Consciousness itself as nondual awareness knows that it is aware without needing mediation by mental representations and without taking itself as an intentional object (Rabjam, 2001; Josipovic, 2019). Nondual reflexivity is the essential property of consciousness itself that makes it what it is (Josipovic, 2019; Josipovic and Miskovic, 2020).

As this awareness is nondual, it cannot not take itself as its object nor can it be something that a separate subject possesses as a capacity. Rather, it knows itself by being itself, through its self-presencing or self-disclosing (Guenther, 1984; Manjusrimitra, 2001). With respect to phenomena, nondual awareness functions like a mirror, merely “mirroring” what is present in experience, without categorizing, labeling, associating, evaluating, deciding, etc. (Rabjam, 2001; Norbu, 2013; Josipovic, 2019; Josipovic and Miskovic, 2020).

Nondual awareness is in itself entirely without both conceptual and non-conceptual representations. Hence, it is entirely silent and unmoving like empty space. It does not make any utterances about itself or anything else. Just as space is more subtle than all things in it, this awareness is also more subtle than all phenomenal contents and global states that co-occur with it (Rabjam, 2001; Josipovic, 2019, 2021; Metzinger, 2024).

The idea that awareness is always an awareness of something, and therefore, necessarily intentional and relational, can be seen as being based on the unconscious semantic structuring of cognition into a subject and object and on the misidentification of foundational nondual awareness with a conceptually reified subject who is attending to and monitoring contents and states (Higgins, 2013; Josipovic, 2014, 2021). Within the aware epistemic space of nondual awareness, which is non-preferential or choice-less, even very subtle effortless monitoring is an intentionality toward optimization. Similarly, however effortless, attending is a selection. These processes are not intrinsic to nondual awareness itself (for a more detailed discussion see Josipovic, 2019).

For nondual awareness, which is in itself homogenous and singular and merely mirrors any contents and states, the immediacy of its knowing does not make any distance for there to be an intentional relation. Since the reflexivity of this awareness is its inherent property, rather than a result of some function, both its reflexivity and its mirroring of contents and states are single nondual experiencing.

Furthermore, within nondual experiencing, the essential properties of nondual awareness such as being, emptiness, and luminosity also appear as the universal properties of phenomena that co-occur with it, in addition to their specific properties (Rabjam, 2001, 2007; Josipovic, 2019, 2021). In this sense, phenomena are not different from nondual awareness within which they occur, and knowing them does not require for this awareness to be intentionally related to something other than itself or to abandon its reflexivity.

Owing to the nondual nature of its reflexivity, nondual awareness cannot be mistaken about itself. However, an inferential *a posteriori* belief, or any learned *a priori* belief, can be mistaken about it. In particular, one can hold a mistaken belief that one has realized nondual awareness when one has not yet. Since this awareness is present in every conscious experience even when only implicit, when any less dualistic state is experienced, one can easily have a sense that this is nondual awareness. Then, upon nondual awareness' reflexivity activating clearly, one retrospectively understands that one was mistaken and yet, paradoxically, also knows that this was that which was aware in every experience. With the practice of “abiding as nondual awareness” over time, this awareness becomes revealed in greater depth in terms of its being unique and distinct from the more subtle layers of perceptual, affective, and cognitive constructs and from the various subtle states of consciousness (Manjusrimitra, 2001; Rabjam, 2001; Josipovic, 2019; Josipovic and Miskovic, 2020).

When explicit, the nondual reflexivity of consciousness itself is very subtle and quiet, empty and immediate, and completely intimate, without distance (Rabjam, 2001; Josipovic, 2019; Metzinger, 2024). The progressive loss of this reflexivity has been identified by certain nondual contemplative traditions as the epistemic cause of the sense of a separate self (Rabjam, 2001, 2007; Higgins, 2013). It progresses in a way described in the following: as the luminosity of nondual awareness intensifies, it first becomes very vivid, outshining, and obscuring its other dimensions. With further intensification, it creates a very subtle sense of self as “one who is aware.” Then, it develops a subtle duality between itself and what is present within its epistemic space, which then becomes its subtle object. This split contains the seed of the subject–object conceptual structure, which eventually replaces the knowing via mirroring with knowing via mental representations and constitutes the loss of awareness' inherent nondual reflexivity—its capacity to directly know itself. It now mistakes itself as a conceptual subject and phenomena as its conceptualized objects. In ordinary experiencing, this foundational conceptual structure is reified with layers of representations, associations, and further re-representations (Guenther, 1984; Metzinger, 2010; Josipovic, 2016, 2021).

Phenomenally, nondual awareness is the most essential self in that it is who or what is conscious or aware in any experience, while the other levels of self, such as proto, core-minimal, and extended self, to the extent that they are conscious, appear to it as its contents. However, because of its nondual way of knowing, this awareness is not a self in the usual sense of self as separate from non-self. Since nondual awareness has no preference for what content or state unfolds within it, it is not the self as the one who is attending, monitoring, or recollecting, prompted by conscious and unconscious motivations. As nondual awareness is complete in itself, its bliss is the final reward, so it has no motivation other than itself. This has been expressed in an old saying that the ultimate goal of all doing is being, and being is fully revealed only in nondual awareness. In encounters with others, nondual awareness mirrors how implicit or explicit this awareness is in another, and expressing this may be experienced by another as helpful, liberating, or merely annoying.

## Discussion

Different theories of reflexivity discussed in this research can be seen not only as different mutually exclusive theories but also as different types of reflexivity determined by the gradient of how implicit or explicit nondual awareness is in any experience, the degree to which consciousness itself is self-evident to itself. When nondual awareness is implicit, consciousness knows itself only indirectly through reified representations of the subject and object, and reflexivity is indirect and dualistic. When it is transitional, reflexivity is less representational and different types of reflexivity can occur, with a progressively decreasing relational distance; therefore, these views can be thought of as versions of qualified monism theories. Finally, when nondual awareness is explicit, reflexivity is fully non-representational and non-relational and self-evident as the property of consciousness itself.

On the view that consciousness itself as nondual awareness is always present in an experience irrespective of how implicit or explicit it is, different types of reflexivity discussed in this research could also be seen as a structure with a gradation of conceptual reifications, from coarse dualistic to very subtle monistic close to consciousness itself. Nondual reflexivity, as the non-conceptual primordial knowing, shines through and is reflected in these layers of conceptualizations as different types of reflexivity.

In light of this, Table 1 can be re-organized as Table 2.

**Table 2:** Reflexivity and representation gradient (see text below).

**Nondual awareness**		**Reflexivity**	**Reification**	**Representation**
Implicit	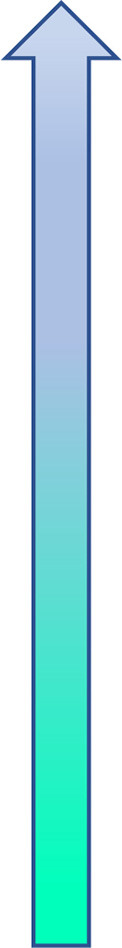	Dual	Coarse	Higher-order reductive conceptual
Transitional		Qualified Monist objectivist	Subtle	Same-order non-reductive conceptual or non-conceptual
		Qualified Monist subjectivist	Very subtle	Same-order or non-representational
Explicit		Nondual	Empty	Non-representational
Radiance of nondual awareness				

Table depicting the “radiating” of nondual awareness through the layers of representations.

When conceptual knowing occurs within explicit nondual awareness, it is both encompassed and pervaded by it, and therefore, it is not different in its essential properties from awareness itself (Norbu, 1987; Josipovic, 2021). At the same time, it retains its relative properties in the hierarchy of concepts, where different types of concepts have differentiable relations to consciousness itself (Singh, 1989; Pruiett, 2016). This is because both the expressions of the nondual authentic being and the expressions of the dualistic self-other concept structure are equally pervaded by the space of nondual awareness.

Some consider phenomenal consciousness to be the great achievement of the evolution of life on earth but the real achievement is much more than mere phenomenality. It is that consciousness has woken up within us and has recognized itself, that within us humans, consciousness knows that it is conscious.

## Author contributions

ZJ: Conceptualization, Writing—original draft, Writing—review & editing.
